# Another Remedy for Asiatic Cholera

**Published:** 1893-10

**Authors:** A. K. Crawford


### ANOTHER REMEDY FOR ASIATIC CHOLERA.

A. K. CRAWFORD, M. D.

When reviewing the therapeutics of Asiatic cholera for the Clinical Society of Hahnemann College last fall, my paper took the form somewhat of a criticism on the homœopathicity of the more common remedies for that disease. The trend and conclusion of the article was, that we have no such thing in our materia medica as a remedy, the pathogenesis of which covers all the symptoms of an attack of cholera. The nearest approach to a picture of that disease was found under Arsenic, but even this drug exhibited certain shortcomings; for instance, the bowel evacuations of Arsenic are either fœcal, or if fluid, they are colored greenish or blackish, and frequently have a putrid odor.

Again, poisoning by *Arsenic* differs from cholera in that it produces a febrile state, while cholera lowers the temperature. The passages of the one are colored and foul, while the others are colorless, odorless, and resemble rice water. There is comparatively little spasm in the pathogenesis of Arsenic while the reverse is the case in the majority of choleraic cases.

The greatest similarity was found in its running in parallel lines with cholera in its capacity to develop a series of stages as cholera does, and because of its profound action on the organism.

*Camphor* was found in no wise to be so close and near a remedy for this malady as Arsenic. In the first place, of twenty-two cases of *Camphor* poisoning cited in the *Cyclopedia of Drug Pathogenesy*, not a man, woman, or child among them died, and a no less notable fact was that beyond its immediate depressing and spasmodic action *Camphor* does not go. The sum of the signs indicating it consisted simply of lividity, algidity, and dyspnœa, so that beyond the very brief period wherein these symptoms occur during the onset of the disease *Camphor* could not be thought of as homœopathic. The grand feature of Asiatic cholera, viz.: the bowel discharges, is altogether absent in the toxic action of this drug.

*Cuprum* displayed as limited applicability to the disease as Camphor. The condition which it meets is that of spasms. Painful contractions and spasmodic action of the voluntary muscles are a marked feature of copper poisoning, but the attempt to make this remedy fit a case of cholera beyond this point was disappointing. *Cuprum* has bowel irritation with choleraic character. There is always increased secretion of bile and the stools are colored in accordance. It irritates more than it inflames. The primary action of Cuprum is on the alimentary mucous membrane, and all the nervous phenomena are secondary to that. The nervous symptoms are entirely irritative, and might be expressed in one word, spasm.

*Veratrum-album* provings supply a symptom which those other drugs lack. It has violent vomiting with continuous nausea and great prostration. It has gushing, profuse rice-water stools. It has tonic cramps of the hands and feet, with icy coldness in these members. The sweat is cold, especially marked on the upper part of the body and forehead. It develops even the sunken hippocratic face.

If we stop here and look over these remedies, which are the most potent ones heretofore used against cholera, we find that Camphor applies to the very first symptoms noted in an attack of cholera; that *Cuprum* is called for in the second set of symptoms in the development of the case, and that Veratrum-alb. fills up the gap of symptoms which neither one covered; while Arsenic from beginning to end runs in somewhat parallel lines, yet not sufficiently close to the disease to make it a true simillimum.

The remedy to which I wish to draw your attention, and which I believe bears a closer homœopathic relationship to Asiatic cholera than any one of the preceding, is the *Agaricus*, or poisonous mushroom. There are some nine different varieties of this fungus of which we have provings in Allen's *Encyclopædia*, all of which are in brief excepting the *muscarius*, which covers fifty-seven octavo pages. They all have strongly marked family traits, but the one which seems to resemble Asiatic cholera most closely is the *Agaricus phalloides*.

The dejections of the *muscarius* are mostly of a pappy nature. The evacuations are frequent and diarrhœic, often semi-fluid, and occasionally cases develop watery stools. There is a repetition in these cases of the symptoms of heaviness in the abdomen, distresses and burning in the anus, and a fullness of the hemorrhoidal veins, and a few cases develop black or bloody stools, running into a dysenteric flux. But let me read to you from the cases of poisoning by the *Agaricus phalloides*, which is a small stinking species of mushroom common in Europe and Pennsylvania.

There is delirium with faint, indistinct dreams, great restlessness, and complete consciousness until death, or there may be stupor. There is death-like palor; face cyanotic, hippocratic countenance, and very anxious expression; cold tongue, cold breath, trismus, inability to answer questions, speech indistinct, violent thirst, nausea, vomiting and diarrhœa, pains in the stomach, abdomen tense, hard, tumid, and painful; frequent bilious stools, frequent watery stools, again whitish stools, as in Asiatic cholera; suppression of urine, voice hoarse, respiration short, pulse small and intermittent, sometimes hardly perceptible; cold extremities, the skin losing its elasticity and assuming a livid hue; cramps of the legs, calves, and feet, which are painful and convulsive; violent general convulsions, which begin in the arms and legs, gradually extending to the muscles of the trunk, causing irregular distortions of the whole body.

Accompanying these symptoms there is extreme prostration, great exhaustion, with restlessness, livid skin, cold sweat, and somnolence. In one well recorded case (in Allen's *Supplement*), the temperature record was slightly below normal; in another the temperature showed a fraction of a degree above.

This poisonous fungus, then, when taken into the economy is sufficiently virulent in its action to cause death, and the picture which it presents, while the subject suffers from its ingestion, is such that all those who have witnessed it, I think, will concur with me that it resembles in a wonderful degree an Asiatic cholera case. It has the lividity, algidity, and dyspnœa as strongly marked as Camphor; it has the spasms, violent convulsions, delirium, and comatose state of Cuprum. It has emesis and diarrhœa, with odorless, watery stools, cold sweat on the face, neck, and chest, and heart failure as prominently as *Veratrum.*

Add to the above facts that it develops a series of stages very similar in their accession, progress, and termination to Asiatic cholera, and we must admit that it deserves to be placed on a higher plane in the therapeutics of this disease than Arsenic itself. We find under this drug the same suddenness of onset of symptoms as is noted in cholera; the same sphere of the system on which it wreaks its vengeance, and the same drift toward a fatal ending. I think I have quoted sufficiently the symptoms, course, and stage of the toxic effects of this drug to prove the point which I claim for *Agaricus*, viz.: that it is and should be considered as one of our most potent remedies wherewith to combat Asiatic cholera.

If it requires anything to supplement its curative influence, it seems to me all that is necessary is to add *Veratrum-alb.*, and this only when the character of the evacuations is persistently of the rice-water variety.

If we are so unfortunate as to be visited by an epidemic of the Asiatic cholera this year, or any other year, I beg of you, brother physicians, to keep this remedy in mind, and to make a trial of it under the laws of similars, and thereby prove its usefulness or uselessness beyond peradventure.—*Medical Century, Vol. I, No.* 8, *August*, 1893.

